# A systematic review of clinical studies on hereditary factors in pelvic organ prolapse

**DOI:** 10.1007/s00192-012-1704-4

**Published:** 2012-03-16

**Authors:** Sabrina L. Lince, Leon C. van Kempen, Mark E. Vierhout, Kirsten B. Kluivers

**Affiliations:** 1 Department of Obstetrics and Gynecology (791), Radboud University Nijmegen Medical Centre, PO Box 9101, 6500 HB Nijmegen, The Netherlands; 2 Department of Pathology, McGill University, Montreal, QC Canada; 3 Lady Davis Institute for Medical Research, Jewish General Hospital, Montreal, QC Canada

**Keywords:** Family, Genetics, Inheritance, Pelvic organ prolapse, Review

## Abstract

**Introduction and hypothesis:**

There is growing evidence that pelvic organ prolapse (POP) is at least partly caused by underlying hereditary risk factors. The aim of our study was to provide a systematic literature review and meta-analysis of clinical studies on family history of POP as a risk factor for POP in individual women.

**Methods:**

The databases PubMed and Embase were searched. Clinical studies reporting on family history of POP in relation to POP in individual women were included.

**Results:**

Sixteen studies were included, of which eight enabled us to calculate a pooled odds ratio (OR). The pooled OR of POP in case of a positive family history of POP was 2.58 (95 % confidence interval 2.12–3.15).

**Conclusions:**

Women with POP are substantially more likely to have family members with the same condition compared to women without POP. This strengthens the hypothesis that genetic predisposition plays an important role in the development of POP.

## Introduction

Pelvic organ prolapse (POP) is a major health care problem with prevalence varying from 8 to 41 % [1, 2] and has a substantial impact on quality of life [3]. Women with POP symptoms not only experience physical complaints, but also encounter more problems regarding general health, personal relationships and sexual function [3].

In order to reduce the number of women developing POP, it is important to know the possible risk and protective factors. The aetiology of POP is considered to be multifactorial. Research groups investigated a large variety of risk factors for POP, such as parity [2, 4–11], body mass index (BMI) [2, 4, 5, 7, 8, 10–13], menopausal state [4, 7–10, 12, 14] and previous hysterectomy [2, 9, 11, 13] with often conflicting results. One of the reasons for these conflicting results might be the fact that not all women are equally susceptible to POP. Some women with multiple risk factors never develop POP, whereas others with little to no risk factors develop POP [15], sometimes already at a young age. Bump and Norton [16] were the first to describe a model for the development of pelvic floor dysfunctions. They divided the different risk factors into predisposing factors (e.g. hereditary factors, collagen distribution), inciting factors (e.g. vaginal delivery), promoting factors (e.g. obesity) and decompensating factors (e.g. aging).

There is growing evidence that predisposing hereditary factors are indeed important in the development of POP. For instance, Buchsbaum et al. [17] found a high rate of concordance between the POP stage of a parous woman and her nulliparous sister, thereby suggesting a familial predisposition toward the development of this disorder. Altman et al. [18] investigated the prevalence of surgical treatment for POP in both dizygotic and monozygotic female twin pairs, knowing that dizygotic twins share approximately 50 % of their DNA, while monozygotic twins are 100 % identical. They found that the influence of genetic factors was substantial, attributing to 40 % of the variation of liability for POP. Analysis of the familial inheritance pattern of POP was performed by Jack et al. [19]. They studied ten families of young women presenting with severe POP. In these families, POP was transmitted in a dominant fashion with an incomplete, but high degree of penetrance, through both maternal and paternal relatives.

A genetic risk for developing POP in members of affected families has important implications for clinical practice. Individual risk assessment for POP and POP recurrence might have consequences for both preventive strategies and the choice of (surgical) treatments.

The aim of our study was to provide a systematic literature review and meta-analysis of clinical studies on family history of POP as a risk factor for POP in individual women.

## Materials and methods

The databases PubMed and Embase were searched in association with a senior librarian up to 1 June 2011. The search terms used for each database included different terms for POP, genetics, inheritance and family and are presented in detail in the Appendices 1 and 2. References of relevant studies were cross-checked for additional studies. Two authors (S.L. and K.K.) evaluated all studies independently. Any disagreement was discussed and resolved in a consensus meeting.

Clinical studies reporting on familial history of POP in a cohort of women with and without POP were included. In case a study only provided information on family history in the POP group, this study was included as an “uncontrolled study”. Publications reporting on patients with symptoms other than POP such as incontinence, rectal prolapse, perineal descent, ureterocele and/or urethral prolapse only were excluded as well as review articles, case reports and studies reporting on less than ten (index) patients. Different studies reporting on the same patient cohort were included once. There were no language restrictions.

In all relevant studies data on study design, study population, sample size, analyses for possible confounders, definition of POP, definition of positive family history and baseline characteristics were collected. The baseline characteristics that were taken into account were age, parity, vaginal deliveries, menopausal status, previous surgery for POP, previous hysterectomy and BMI. Most importantly, data on positive family history of POP in relation to absence or presence of POP in the (index) patient were collected. In case an article did not provide the above-mentioned information, authors were asked to supply the data needed within an acceptable time frame. In case of missing data with regard to family history in relation to individual POP status, these data were excluded from final analysis.

The number of patients with a positive family history in both POP and control groups was used to calculate the crude odds ratio (OR) of each study. The pooled OR with the 95 % confidence interval (CI) is presented. The heterogeneity index (I^2^) was used to measure the inconsistency between the studies [20].

## Results

### Results of the search

**Fig. 1:**
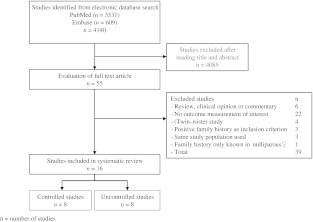
Database search flow chart

The PubMed search revealed 3,531 studies, and the Embase search revealed an additional 609 studies. No studies were revealed by cross-checking; 4,085 of these studies could be excluded on the basis of title and/or abstract. The remaining 55 studies were read by paper. In total 16 studies fulfilled the inclusion criteria. The reasons for excluding the other 39 studies are indicated in Fig. 1. The (twin) sister studies were excluded because although these studies evaluated POP in the (twin) sister, they did not investigate the presence of POP in all sisters. The results of these studies were therefore not a reliable representation of the family history.

### Study design and participants

In total 4,354 participants were included in this meta-analysis and review: 2,413 women with POP and 1,941 without POP (Tables 1 and 2). Of the 2,413 cases with POP, 1,107 could be included in the meta-analysis. The remaining 1,306 were included as uncontrolled studies.

**Table 1:** Study characteristics of the included controlled studies on family history of POP in women with and without POP

First author (year)	Study design	Number of participants^a^ and characteristics	Age in years^b^	Parity^b^	Vaginal deliveries^b^	BMI in kg/m^2 b^	Postmenopausal	Previous POP surgery	Previous hysterectomy	Definition positive family history	% (*n*) of patients with positive family history
Braekken (2009) [21]	Matched case-control study	49 cases with POP-Q≥ stage II	47.3 (±11.2)	2 [1–5]	*n* = 43 (87.8 %) only vaginal deliveries	24.9 (±3.8) in both groups together	*n* = 20 (56 %)	Not reported	*n* = 4	Mother or grandmother with pelvic floor disorder	49 (24)
		49 controls with POP-Q≤ stage I	47.0 (±10.6)	2 [1–5]	*n* = 40 (81.6 %) only vaginal deliveries		*n* = 16 (33 %)		*n* = 2		33 (16)
					no women with only SC						
Chiaffarino (1999) [4]	Case-control study	76 (108) cases with Baden-Walker grade 2 or 3 uterovaginal prolapse and/or grade 3 cystocele	*n* = 46 (43 %) ≤56	*n* = 5 (5 %) 0	*n* = 5 (5 %) 0	*n* = 37 (35 %) ≤23	*n* = 85 (81 %)	Not reported	Not reported	Mother with POP	28 (21)
			*n* = 31 (29 %) 57–64	*n* = 24 (22 %) 1	*n* = 26 (24 %) 1	*n* = 32 (31 %) 24–26					
			*n* = 31 (29 %) ≥65	*n* = 79 (73 %) ≥2	*n* = 77 (71 %) ≥2	*n* = 36 (34 %) >26					
		85 (100) controls with no POP or UI, but other acute, non-gynaecological, non-neoplastic conditions	*n* = 35 (35 %) ≤56	*n* = 17 (17 %) 0	*n* = 17 (17 %) 0	*n* = 34 (34 %) ≤23	*n* = 88 (88 %)				11 (9)
			*n* = 30 (30 %) 57–64	*n* = 28 (28 %) 1	*n* = 28 (28 %) 1	*n* = 28 (28 %) 24–26					
			*n* = 35 (35 %) ≥65	*n* = 55 (55 %) ≥2	*n* = 55 (55 %) ≥2	*n* = 37 (37 %) >26					
McLennan (2008) [32]	Cohort study	194 cases with Baden-Walker grade 1–4 POP	Not reported	Not reported	Not reported	Not reported	Not reported	Not reported	*n* = 82 (100 %)	POP and/or hernia in family	71 (137)
		264 controls without POP							*n* = 124 (100 %)		47 (124)
Miedel (2009) [33]	Case-control study	252 (273) cases with symptomatic POP	53.3 (±12.3)	1 [0–5]	CS rate 13.0 %	*n* = 8 (3 %) <20	Not reported	*n* = 0 (0 %)	Not reported	Mother or sister with POP	20 (51)
						*n* = 128 (47 %) 20–24					
						*n* = 102 (38 %) 25–29					
						*n* = 33 (12 %) ≥30					
		264 (285) controls without POP complaints	49.1 (±13.5)	1 [0–5]	CS rate 8.6 %	*n* = 18 (6 %) <20		*n* = 0 (0 %)			9 (23)
						*n* = 181 (64 %) 20–24					
						*n* = 65 (23 %) 25–29					
						*n* = 18 (6 %) ≥30					
Rodrigues (2009) [8]	Case-control study	107 cases with POP-Q stage III or IV	Mean 66.3	Mean 4.5	Mean 4.0	Mean 27.8	*n* = 107 (100 %)	Previous vaginal surgery was exclusion criterion	Not reported	First-degree relatives with POP	28 (30)
		209 controls with POP-Q stage 0 or I	Mean 60.8	Mean 2.0	Mean 1.0	Mean 26.5	*n* = 209 (100 %)				17 (36)
Sewell (2007) [31]	Cohort study	73 cases with POP-Q stage II–IV and/or previous POP surgery	45.8 (±12.4)	2.2 (±1.7)	2.0 (±1.8)	Body weight 62.3 kg (±11.5)	*n* = 22 (30 %)	*n* = 4 (5.4 %)	*n* = 8 (11.0 %)	Not reported	11 (8)
		93 (94) controls with POP-Q stage 0 or I and without previous POP surgery	44.5 (±14.3)	1.3 (±1.5)	1.0 (±1.4)	Body weight 70.5 kg (±20.4)	*n* = 28 (30 %)	*n* = 0 (0 %)	*n* = 4 (4.3 %)		8 (7)
Sharma (2003) [24]	Matched case-control study	225 cases with self-reported POP	Not reported	Not reported	Not reported	Not reported	Not reported	Not reported	Not reported	Not reported	11 (24)
		225 controls without self-reported POP									1 (3)
Slieker-ten Hove (2008) [7]	Cohort study	131 (213) cases with symptomatic POP and/or previous POP surgery	60.6 (±9.8)	2.4 (±1.2)	CS rate 5.1 %	25.9 (±3.5)	*n* = 120 (56 %)	*n* = 95 (45 %)	*n* = 36 (22.6 %)	Mother with POP	56 (73)
		752 (1037) controls without POP complaints or previous POP surgery	57.2 (±8.8)	2.3 (±1.7)	CS rate 4.7 %	25.6 (±3.8)	*n* = 518 (50 %)	*n* = 0 (0 %)	*n* = 191 (15.9 %)		34 (252)

Total POP *n* = 1,107 (1,242); total controls *n* = 1,941 (2,263)

*n* number of patients, *POP-Q* Pelvic Organ Prolapse Quantification system, *POP* pelvic organ prolapse, *UI* urinary incontinence, *SC* caesarean section

^a^Total number of participants if different from number of participants answering question on family history

^b^Data represented as mean (± standard deviation) or median [range] if not stated otherwise

**Table 2:** Study characteristics of included uncontrolled studies on family history of POP in women with POP

First author (year)	Study characteristics	Number of participants^a^ and characteristics	Age in years^b^	Parity^b^	Vaginal deliveries^b^	BMI in kg/m^2^ ^b^	Postmenopausal	Previous POP surgery	Previous hysterectomy	Definition positive family history	% (*n*) of patients with positive family history
Deval (2002) [25]	Case-control study	37 cases with POP surgery for POP grade 2 (Baden-Walker)	38.2 (±4.2)	2.8 (±1.0)	*n* = 1 (3 %) with SC	25.5 (±3.8)	Not reported	Not reported	Not reported	Not reported	32 (12)
Forsgren (2008) [26]	Case-control study	90 hysterectomized women undergoing vaginal vault surgery	72.8 (±9.7)	2.3 (±1.1)	*n* = 86 (96 %)	25.0 (±3.7)	100 %	Not reported	100 %	Not reported	32 (29)
Jeon (2008) [27]	Cohort study	212 cases with POP surgery for POP-Q stage ≥II	59.3 (±9.1)	3.7 (±1.7)	*n* = 210 (99 %)	24.3 (±2.6)	*n* = 184 (87 %) postmenopausal	*n* = 18 (9 %)	Not reported	Mother or sister with POP	21 (44)
Lukanovic (2010) [22]	Matched case-control study	82 cases with surgical repair of vaginal cuff prolapse below the hymenal remnants after hysterectomy for benign pathology	52.2 (±9.5)	Not reported	2.3 (±1.1)	Body weight 68.7 kg (±10.5)	Not reported	POP as indication for hysterectomy *n* = 46 (56.1 %)	100 %	Not reported	41 (34)
Nava y Sánchez (1980) [28]	Cohort study	50 cases with uterine prolapse stage II/III	All patients <35	4.5	Not reported	Not reported	Not reported	Not reported	0 %	Not reported	28 (14)
Rechberger (2010) [29]	Cohort study	717 cases scheduled for reconstructive surgery for POP	61.3	1.2 % 0	97 %	27.6	>80 % postmenopausal	15 %	17 %	Grandmother, mother or sister with POP	13 (96)
				24.4 % 1							
				48.2 % 2							
				24.8 % 3							
Rinne (1999) [23]	Matched case-control study	66 (85) cases with at least ≥grade 2 POP, all operated for POP	38.5 (±4.2)	2.8 (±1.0)	Not reported	25.5 (±3.8)	Not reported	Not reported	Not reported	At least one degree relative with POP	30 (20)
Shilo (2009) [30]	Cohort study	52 cases operated for POP	*n* = 26 <45	Not reported	Not reported	Not reported	Not reported	Not reported	Not reported	First-degree relatives with POP	27 (14)
			*n* = 26 >55								

Total POP *n* = 1,306 (1,325)

*n* number of patients, *POP-Q* Pelvic Organ Prolapse Quantification system, *POP* pelvic organ prolapse

^a^Total numbers of participants if different from number of participants answering question on family history.

^b^Data represented as mean (± standard deviation) or median [range] if not stated otherwise

The 16 included studies consisted of 7 cohort studies and 9 case-control studies (Tables 1 and 2). Four of the case-control studies were designed as matched case-control studies, matching for age [21–24], parity [21, 22, 24], menopausal status [22], hospital at which hysterectomy was performed [22], year of surgery [22], indication [22] and type of surgery [22]. In two of the nine case-control studies [23, 25], only the women with POP were asked whether they had relatives with POP. Therefore, only the women in the POP group were included for analysis in this review. In two of the case-control studies [22, 26] the control group contained some women with previous POP surgery. Since this could not be regarded as a valid control group, the studies were classified as uncontrolled. Four cohort studies [27–30] did not include a control group and only reported on women with POP. In all other studies both women with and without POP were asked whether they had a positive family history of POP. Two studies only included women with a previous hysterectomy [22, 26].

### Definition of POP

The definition of POP varied between studies (Tables 1 and 2); four studies used the Pelvic Organ Prolapse Quantification (POP-Q) system of which two defined POP as POP-Q stage ≥II [21, 27] and one as POP-Q stage III/IV [8]. One study provided information on all different POP-Q stages [31]. We decided to choose POP-Q stage ≥II as a cut-off point. Three studies used the Baden-Walker halfway scoring system; one defining POP as Baden-Walker grade 2 [25], one as ≥grade 1 [32] and one as second- or third-degree uterovaginal prolapse and/or third-degree cystocele [4]. Three other studies did not mention any classification system but used the terms “below the hymenal remnants” [22], “≥ grade 2” [23] and uterine prolapse stage II/III [28] without referring to any grading system. In six studies, the POP group consisted only of women who had either undergone POP surgery or were scheduled for it [22, 23, 25–28]. Three of these studies used POP surgery or vaginal vault surgery as the definition of POP, without further defining the severity of POP [26, 29, 30]. In addition three studies used symptoms as assessed with questionnaires to identify women with POP [7, 24, 33].

### Definition of family history

The definition of family history was not homogeneous throughout the studies either; two studies included both mother and sisters in the family history [27, 33], one study included mother and grandmothers [21], one study mother, grandmothers and sisters [29], two studies only the mother [4, 7] and two studies all first-degree family members [8, 30].

Six studies did not specify the degree of relationship [22, 24–26, 28, 31]. A special remark should be made regarding the study of McLennan et al. [32], as they also included male and female relatives with a history of hernia. With the data provided, we were not able to recalculate the number of women with only family members affected with POP.

### Baseline characteristics

Baseline characteristics of the 16 included studies are outlined in Tables 1 and 2. Table 1 presents baseline characteristics of the controlled studies and Table 2 of the uncontrolled studies.

### Statistical power

Only three studies [21, 26, 33] reported that a power analysis was performed for sample size calculation. The power calculation of the study of Braekken et al. [21], however, was based on the expected difference in joint hypermobility.

### Confounders

Except for the study of Sewell et al. [31] all individual studies considered possible confounding factors, either through statistical analyses or through study design.

### Results on family history of POP

**Fig. 2:**
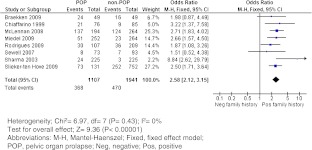
Forest plot of the eight controlled studies

The (crude) ORs of the eight controlled studies are presented in a forest plot (Fig. 2). All but two studies [21, 31] showed a statistically significant difference in the prevalence of positive family history of POP between women with POP and women without this condition.

Eight studies [23, 25–30] only investigated family history in women with POP. Therefore, no OR could be calculated. The number and percentages of women with a positive family history in these studies are shown in Table 2.

The overall OR of POP in case of a positive family history of POP as compared to a negative family history was 2.58 (95 % CI 2.12–3.15). Note that the study of McLennan et al. [32] also included male and female relatives with a history of hernia. When excluding this study, the OR was slightly different (2.54; 95 % CI 2.02–3.20).

Finally, we also calculated the pooled OR, using the adjusted OR if presented in the individual studies (data not shown). By using these data, we found that the pooled OR was lower but still statistically significantly larger than 1.

### Results for family history and recurrences of POP

One study [27] analysed risk factors for the recurrence of POP in 212 women undergoing surgery for symptomatic POP. After a median follow-up of 24 months (range 1–84 months), 36 patients (17 %) presented with recurrence of POP. After correcting for other possible risk factors, such as age, parity, mode of delivery and BMI, family history was not found to be a risk factor for the recurrence of POP. Only preoperative POP-Q stage IV appeared to be associated.

## Discussion

### Summary of main results

This paper summarizes the available literature on family history of POP women with and without POP. All but two studies [21, 31] found a statistically significant difference in the prevalence of positive family history between women with POP and women without this condition. A meta-analysis of these studies resulted in a substantially increased risk for POP in case of a positive family history. In conclusion, there is strong evidence that women with at least one female family member with POP have a significantly higher risk of developing POP.

This suggests an underlying genetic susceptibility. Over recent years, different research groups have been looking for possible gene candidates, by searching for DNA polymorphisms associated with POP. A number of DNA polymorphisms associated with POP have been identified so far: laminin γ1 [34], estrogen receptor α and β [35, 36], progesterone receptor [37], collagen, type III, alpha 1 [38–41] and matrix metalloproteinase-9 [42]. However, not all studies find the same associations. Jeon et al. [40] found that the GG genotype of the collagen, type III , alpha 1 (COL3A1) polymorphism was significantly more prevalent among Korean women with POP, whereas both Kluivers et al. [38] and Chen et al. [39] concluded that the AA genotype was significantly associated with POP in Caucasian and Taiwanese females, respectively. On the other hand, Martins et al. [41] found no correlation between the COL3A1 polymorphism and POP in their population. As was already suggested by Martins et al. [41], these differences could be explained by the fact that all studies were performed in ethnically different populations, i.e. Korean, Dutch, Taiwanese and Brazilian, with different background risks of POP.

Only one study so far looked at family history and the risk of recurrence of POP. If there is an underlying hereditary factor causing weaknesses in the pelvic floor, e.g. by changes in collagen strength, this might lead to impaired healing properties after surgery and therefore to more recurrences. If this is the case, first-line treatment with mesh material might be indicated for patients with positive family history. The study of Jeon et al. [27], however, did not find such an association and only found preoperative POP-Q stage IV to be a risk factor for recurrence. This last finding is in line with the study of Salvatore et al. [43], who confirmed that preoperative stage ≥III was the only risk factor for recurrences.

### Uncontrolled studies

As for the studies not included in the meta-analysis, the mean percentage of POP patients with a positive family history was 28 %. This is comparable with the mean percentage of the controlled studies. It is notable that three of the studies [23, 25, 28] in the uncontrolled group consist of rather young women less than 40 years of age. One could hypothesize that when young women develop POP, there must be an underlying hereditary factor causing early decompensation of the pelvic floor. It might therefore be possible that those young women more often have family members with POP. The above-mentioned studies, however, do not show an increase in positive family history among these young women. This is in contrast with the study of Shilo et al. [30] that shows a more than fivefold increase in positive family history among women under the age of 45 years as compared to women above the age of 55.

### Strengths and limitations

The strength of this review is that all articles on family history and POP were taken into account, without any language limitations. This resulted in a complete overview of studies in ethnically diverse populations, from the European, Asian and North and South American continents. It is therefore reasonable to suggest that the observed hereditary susceptibility is present in women worldwide, irrespective of possible population-dependent background risks. Nevertheless, it remains possible that different DNA polymorphisms are responsible for the increased risk of POP among women of different ethnic descent.

In total eight studies could be used for the meta-analysis. Since these are all the studies available at the moment, we feel that we are drawing our conclusions on the best available evidence. Moreover, all studies point in the same direction, which makes it unlikely that additional research will alter our results drastically.

As can be seen in Table 1, baseline characteristics of the studies were not totally similar. In most studies, the mean parity was 1 to 2. However, the women in the POP group of the study of Rodrigues et al. had a mean of 4.5 deliveries. Besides, in some studies the women included were clearly younger than in other studies. Of course, this also had its influence on menopausal status.

Likewise, the design of the studies was not always identical; five of the eight studies were case-control studies, whereas the other three were cohort studies. Furthermore, the definition of the POP and control groups differed between studies.

Regarding the statistics used, we found no statistical heterogeneity between the studies (*I* ^2^ = 0; *p* = 0.47, χ^2^ for heterogeneity); therefore, the pooled OR is presented.

As already said, the studies were not homogeneous regarding their definition of POP. Most studies used anatomical landmarks to divide women into case and control groups. Some however chose more strict criteria, e.g. only included women with POP-Q stage III or IV urogenital prolapse in the case group and women with POP-Q stage 0 or I in the control group [8]. It seems plausible that by applying more strict criteria, the difference in prevalence of positive family history between groups would be more pronounced. However, this was not depicted in our meta-analysis.

Three studies [7, 24, 33] used questionnaires to define the presence or absence of POP. Symptoms of POP, however, do not always fully correspond to the existence of anatomical POP. For example, in the study of Samuelsson et al. [11] 10 % of the women with POP reported a “sense of heaviness in the lower abdomen” compared to 8 % of the women without anatomical POP, a difference that was not statistically significant. Barber et al. [44] performed a study to identify the symptoms that most accurately predicted the presence or absence of POP. They found that the question “Do you usually have a bulge or something falling out that you can see or feel in your vaginal area” had the best discriminative capability. The study of Tan et al. [45] confirmed that there was a significant relationship between symptoms of vaginal bulging and POP on gynaecological examination. The studies by Miedel et al. [33], Sharma et al. [24] and Slieker-ten Hove et al. [7] used this question for discriminative purposes and were therefore included in this review.

The definition of family history also varied between studies. It is likely that in studies using broader criteria, more women will report a positive family history. However, this is likely to be the same for both POP patients and controls. Therefore, this will not necessarily introduce bias.

Except for three studies [7, 24, 33], all studies were performed among women visiting an outpatient department for regular check-up, prolapse complaints or other benign gynaecological conditions. These results therefore may not be generalizable to the general population. However, the results of the two cohort studies conducted in the general population [7, 33] and the population-based case-control study [24] show the same results. It seems therefore plausible that both in low- and high-risk populations positive family history is an important risk factor for the development of POP.

Recall bias might play a role in the differences found between patients and controls. Because POP patients are more focused on POP complaints, they may be more aware of similar complaints in relatives and therefore more often report a positive family history compared to controls. However, evidence for recall bias was absent in comparable studies on family history of myocardial infarction and colorectal cancer [46, 47]. The controls in these studies reported family history in first- and second-degree relatives as accurately as the patients did.

Finally, regarding possible publication bias, it needs to be noted that we only used published studies and abstracts for our review and meta-analysis. We performed a funnel plot and found no deviation from a symmetrical shape. Publication bias seems therefore unlikely.

### Authors’ conclusions

This meta-analysis of 8 studies with 1,107 POP patients and 1,941 controls showed that women with POP are substantially more likely to have family members with the same condition compared to women without POP. This demonstrates that a positive family history is an important risk factor for POP. The fact that the uncontrolled studies showed a high percentage of positive family history in POP patients further underlines this conclusion. It strengthens the hypothesis that genetic predisposition plays an important role in the development of POP.

This information is important for clinical practice from a preventive point of view. Although not all known risk factors for POP may be modifiable, risk factors such as obesity [2, 10, 12] and heavy physical work [7] are. In addition, pelvic floor physiotherapy might reduce the risk of developing POP. Although statistical evidence that (peripartum) pelvic floor exercises can decrease the risk of developing POP later in life is lacking [48], it is known that pelvic floor muscle function is significantly related to POP [21]. Furthermore, a recent study showed that pelvic floor muscle training supervised by a physical therapist leads to an increased volume of the pubovisceral muscle, a decreased hiatal area and an elevated resting position of the bladder and rectum [49]. This suggests that pelvic floor physiotherapy can play a role in primary and secondary prevention of POP and might therefore be of great importance in women with an increased risk of POP.

Vaginal delivery can have a detrimental effect on pelvic floor muscle strength and could thus promote POP. Although there are some studies reporting an association between caesarean section and a reduced prevalence of POP [50, 51], the preventive value of a primary caesarean section is unproven. A recent large study [52] did not find a significant difference between frequency of POP 20 years after caesarean or vaginal birth. Since a caesarean section is an invasive procedure with potentially substantial risks for the mother, we do not advocate this until there is more evidence of a protective effect available.

In conclusion, we summarized the evidence available on family history as a risk factor for the development of POP. Our primary conclusion was that there is a substantially increased risk for POP in case of a positive family history. This knowledge might influence the way patients with a positive family history could be treated. From a preventive point of view, patients could be informed about risk factors and how to try to avoid them and could be advised to perform pelvic floor exercises before problems arise. Until now, there is no evidence that vaginal delivery should be discouraged. On theoretical grounds, first-line treatment with mesh material might be indicated for patients with positive family history. To the best of our knowledge, no studies on this topic have been conducted so far, so this hypothesis should be the subject of future research.

## Appendix 1. Full PubMed literature search terms

(("genetics"[MeSH Terms] OR "genetics"[Subheading] OR "Genetic Predisposition to Disease"[Mesh] OR genetic* OR inheritance OR family OR families OR familial OR gene OR genes OR DNA)) AND ("Pelvic Organ Prolapse"[Mesh] OR ((pelvic OR vaginal OR uterus OR bladder OR rectum OR rectal OR bowel OR vagina OR urethra OR perineal OR perineum OR uterine OR cervix OR cervical OR vault OR cuff OR genital OR urogenital) AND (descent OR prolapse)) OR uterine prolapse OR rectal prolapse OR cystocele OR rectocele OR enterocele OR proctocele OR sigmoidoceles OR peritoneocele OR ureterocele OR cystourethrocele OR cysto-urethrocele OR pelvic floor dysfunction).

## Appendix 2. Full Embase literature search terms

exp genetics/ or genetics.fs. or exp genetic predisposition/ or (genetic* or inheritance or family or families or familial or gene or genes or DNA ).mp. AND exp pelvic organ prolapse/ or ((pelvic or vaginal or uterus or bladder or rectum or rectal or bowel or vagina or urethra or perineal or perineum or uterine or cervix or cervical or vault or cuff or genital or urogenital).mp. AND (descent or prolapse).mp.) or (uterine prolapse or rectal prolapse or cystocele or rectocele or enterocele or proctocele or sigmoidoceles or peritoneocele or ureterocele or cystourethrocele or cysto-urethrocele or pelvic floor dysfunction).mp.
